# STAT3 Signalling via the IL-6ST/gp130 Cytokine Receptor Promotes Epithelial Integrity and Intestinal Barrier Function during DSS-Induced Colitis

**DOI:** 10.3390/biomedicines9020187

**Published:** 2021-02-12

**Authors:** Lokman Pang, Jennifer Huynh, Mariah G. Alorro, Xia Li, Matthias Ernst, Ashwini L. Chand

**Affiliations:** 1 Olivia Newton-John Cancer Research Institute, La Trobe University School of Cancer Medicine, Heidelberg, VIC 3084, Australia; Jennifer.huynh@onjcri.org.au (J.H.); mariah.alorro@onjcri.org.au (M.G.A.); matthias.ernst@onjcri.org.au (M.E.); 2 Department of Mathematics and Statistics, La Trobe University, Bundoora, VIC 3083, Australia; x.li2@latrobe.edu.au

**Keywords:** STAT3, IL-6ST/gp130, colitis, inflammation, intestinal barrier function

## Abstract

The intestinal epithelium provides a barrier against commensal and pathogenic microorganisms. Barrier dysfunction promotes chronic inflammation, which can drive the pathogenesis of inflammatory bowel disease (IBD) and colorectal cancer (CRC). Although the Signal Transducer and Activator of Transcription-3 (STAT3) is overexpressed in both intestinal epithelial cells and immune cells in IBD patients, the role of the interleukin (IL)-6 family of cytokines through the shared IL-6ST/gp130 receptor and its associated STAT3 signalling in intestinal barrier integrity is unclear. We therefore investigated the role of STAT3 in retaining epithelial barrier integrity using dextran sulfate sodium (DSS)-induced colitis in two genetically modified mouse models, to either reduce STAT1/3 activation in response to IL-6 family cytokines with a truncated *gp130^∆STAT^* allele (GP130^∆STAT/+^), or by inducing short hairpin-mediated knockdown of *Stat3* (shStat3). Here, we show that mice with reduced STAT3 activity are highly susceptible to DSS-induced colitis. Mechanistically, the IL-6/gp130/STAT3 signalling cascade orchestrates intestinal barrier function by modulating cytokine secretion and promoting epithelial integrity to maintain a defence against bacteria. Our study also identifies a crucial role of STAT3 in controlling intestinal permeability through tight junction proteins. Thus, therapeutically targeting the IL-6/gp130/STAT3 signalling axis to promote barrier function may serve as a treatment strategy for IBD patients.

## 1. Introduction

Inflammatory bowel disease (IBD), including Crohn’s disease (CD) and ulcerative colitis (UC), is characterised by chronic and relapsing inflammation of the gastrointestinal tract [1]. The prevalence of IBD has dramatically increased over the last two decades, with 6.8 million cases diagnosed worldwide in 2017 alone [2]. Moreover, patients with IBD are further predisposed to developing colorectal cancer (CRC), which is the third most common fatal malignancy worldwide [3]. In the gastrointestinal tract, intestinal epithelial cells (IECs) build a physical and biochemical barrier that segregates the intestinal microbiota and the underlying mucosal immune system to maintain homeostasis. Disruption of this barrier leads to increased intestinal permeability and allows the invasion of pathogenic microorganisms. Barrier dysfunction is associated with inflammation and a variety of diseases, including IBD and cancer [4]. However, it remains controversial whether dysfunction in the intestinal epithelial barrier acts as the initial driver for IBD or is of consequence to chronic inflammation.

The Signal Transducer and Activator of Transcription-3 (STAT3) is responsible for mediating cytokine and growth factor signalling in a range of tissues [5]. The most important upstream activators of STAT3 include cytokines of the interleukin (IL)-6 family (i.e., IL-6, IL-11) and the IL-10 family (IL-10, IL-22). In particular, members of the IL-6 family signal through the shared IL-6ST/gp130 receptor to trigger activation of the associated Janus kinase (JAK), which mediates subsequent phosphorylation and activation of STAT3. Once activated, STAT3 translocates to the nucleus to modulate transcription of the target genes involved in cell proliferation, migration, differentiation, survival, and inflammatory responses. However, polymorphisms of JAK/STAT3 pathway genes (i.e., *JAK2, TYK2*, and *STAT3*) or upstream STAT3 activators (*IL-6, IL-11, IL-22, IL6-ST/GP130*) confer susceptibility for IBD including CD and UC [6,7,8]. While it remains unclear how these factors contribute to disease susceptibility, persistent activation of STAT3 is frequently observed in the IECs of human patients with CD and UC [9]. In addition, aberrant STAT3 activation is associated with malignant transformation and pathogenesis of the majority of solid cancers, including colitis-associated CRC and gastric cancer [10,11,12]. Interestingly, mice with *Stat3* ablation in macrophages and neutrophils develop spontaneous enterocolitis [13,14], whereas T-cell specific *Stat3* deletion offers protection against inflammation in a T-cell transfer model of colitis [15]. Similarly, mice with conditional deletion of *Stat3*, in IECs specifically, display increased susceptibility to both dextran sulfate sodium- (DSS) and *Citrobacter rodentium*-induced colitis [16,17]. These results suggest that STAT3-mediated regulation of intestinal immune homeostasis is cell-type dependent. However, the significance as well as the cellular mechanisms by which IL-6ST/gp130/STAT3 signalling promote barrier integrity during DSS-induced colitis are yet to be elucidated. We hypothesised that systemic reduction in STAT3 activity would exacerbate the development of acute colitis injury through the modulation of intestinal barrier integrity.

In the present study, we reduced STAT3 activity in two mouse models by either reducing STAT3 activation with a truncated gp130 receptor or inducing a short hairpin-mediated *Stat3* knockdown. Upon exposure to DSS, IL-6 was abundantly produced in mice with reduced STAT3 activity. We demonstrate that the reduction in STAT3 activity significantly increased susceptibility to DSS-induced colitis, which was manifested by severe weight loss, increased inflammation, and decreased intestinal barrier function. Impaired barrier function was accompanied by dysregulated expression of the antimicrobial genes *Reg3b* and *Reg3g*, as well as decreased expression of the tight junction proteins claudin 2 and 3. Our study indicates that IL-6ST/gp130/STAT3 signalling is critical in promoting intestinal barrier function and epithelial regeneration during colitis.

## 2. Materials and Methods

### 2.1. Animals

GP130^∆STAT/+^, CAG-rtTA3;TRE-Stat3 (shStat3), and CAG-rtTA3;TRE-Luc (shLuc) mice were derived as previously described [18,19]. GP130^∆STAT/+^ mice were maintained on a mixed C57BL/6 × 129SV background, whereas CAG-rtTA3;TRE-Stat3, CAG-rtTA3;TRE-Luc, and RosaCre^ERT2^;Stat3^fl/fl^ mice were maintained on a C57BL/6 background and under specific pathogen-free conditions. Animals were housed in individually ventilated cages (IVC) with a 12:12 h light:dark cycle. Age- and gender-matched mice were used for all experiments. All animal experiments were conducted in accordance with the Animal Ethics Committee of Austin Health (Approval number: A2016/05325; approved 1 May 2016 and A2018/05583; approved 21 November 2018).

### 2.2. DSS-Induced Colitis

Acute experimental colitis was induced in mice by supplementing drinking water with 3.5% DSS (MW = 36–50 kDa; MP Biochemicals, Santa Ana, CA, USA) *ad libitum* until the experimental endpoint, as determined by a 20% loss of initial bodyweight. Mice in the control cohorts received normal drinking water without DSS. To induce the corresponding short hairpin activity, shStat3 and shLuc mice received additional pre-treatment with doxycycline via food pellets (600 mg/kg, Specialty feeds, Australia) for 9 days prior to DSS treatment. In order to induce Cre-recombinase-mediated *Stat3* deletion, RosaCre^ERT2^;Stat3^fl/fl^ mice received two oral gavages of tamoxifen (5 mg/25 g, 60 mg/mL in sunflower oil) 5 days prior to DSS treatment. Mice were monitored daily for weight loss and signs of disease.

### 2.3. Tissue Collection

The entire colon was excised and its length was measured from the ileocecal junction to the anal verge as an indicator of inflammation and injury. The 0.5 cm proximal piece was frozen for RNA analysis and the 0.5 cm distal piece was frozen for protein analysis. The rest of the colon was fixed for 24 h in 10% neutral buffered formalin as a “Swiss roll” then transferred to 80% ethanol. Colons were processed for paraffin embedding and sectioned at 5 µm for staining with H&E, Alcian blue/periodic acid-Schiff staining, or immunohistochemistry.

### 2.4. In Vivo Intestinal Permeability Assay

Intestinal permeability was determined as previously described [20,21]. Briefly, mice were fasted for 16 h at the experimental endpoint, then gavaged with 600 mg/kg body weight of 50 mg/mL FITC dextran (4 kDa, Sigma Aldrich, St. Louis, MO, USA) or 40 mg/mL TRITC dextran (4.4 kDa, Sigma Aldrich, St. Louis, MO, USA). Blood was collected via cardiac puncture 4 h later and serum was isolated. A standard curve was prepared using serial dilutions of dextran in PBS. Fluorescence emission was measured on a SPECTROstar Nano microplate reader (BMG Labtech, Melbourne, Australia) at an excitation wavelength of 490 nm and an emission wavelength of 530 nm for FITC-dextran, or an excitation wavelength of 550 nm and emission wavelength of 575nm for TRITC-dextran.

### 2.5. Gene Expression Analysis

Total RNA was isolated from colonic tissues using a Qiagen RNeasy Plus Mini Kit and reverse transcribed into cDNA using a High Capacity cDNA Reverse Transcription Kit (Applied Biosystems, Foster City, CA, USA) as per the manufacturer’s instructions. Quantitative real-time PCR analysis was performed in duplicate using a SensiFAST SYBR kit (Bioline, Cincinnati, OH, USA) and a Viia7 Real-Time PCR system (Thermofisher, Waltham, MA, USA). Relative expression was calculated using the ∆Ct method and normalised against the house-keeping gene, *Gapdh*. Primers were sourced from Integrated DNA Technologies and the sequences are listed in supplementary data Table S1.

### 2.6. Western Blot

Colonic tissues were lysed in RIPA lysis buffer supplemented with protease and phosphatase inhibitors (Roche, Basel, Switzerland) and homogenized using TissueLyser II (Qiagen, Hilden, Germany). Twenty micrograms of protein was loaded in a 4–12% Bis-Tris polyacrylamide gel and transferred to PVDF membranes using the iBlot Gel Transfer Device (Thermo Fisher Scientific, Waltham, MA, USA). The membranes were blocked with Odyssey blocking buffer (TBS; Li-COR, Lincoln, NE, USA) for 1 h at room temperature. Membranes were incubated overnight at 4 °C with primary antibodies against pSTAT3 (Cell Signalling Technology, Danvers, MA, USA; #9145; 1:500), STAT3 (Cell Signalling Technology, Danvers, MA USA; #4904; 1:1000), claudin 2 (Abcam, Cambridge, UK; #ab53032; 1/1000), claudin 3 (Abcam, Cambridge, UK; #ab15102, 1/500), β-actin (Abcam, Cambridge, UK; #ab8227), and GAPDH (Sigma Aldrich, St. Louis, MO, USA; #G8795; 1/2500). Membranes were incubated with the appropriate secondary antibodies at room temperature for 1 h, then visualised using the Odyssey Infrared Imaging system (LI-COR Biosciences, Lincoln, NE, USA).

### 2.7. Histology and Immunohistochemistry

For goblet cell staining, mouse colonic sections were stained with 1% *w/v* Alcian blue (3% acetic acid), followed by 0.5% *v/v* periodic acid Schiff (PAS) and Schiff’s reagent prior to counterstain with haematoxylin. The percentage of PAS/Alcian blue positive goblet cells was evaluated from at least 10 longitudinally sectioned crypts. Quantification was performed using a modified positive pixel count algorithm with Aperio ImageScope v12.4.0.5043 software (Leica Microsystems Pty Ltd., Wetzlar, Germany). For immunohistochemistry, tissue sections were deparaffinised, rehydrated, and heated in a microwave pressure cooker with 0.1% citrate buffer, pH 6.0. Sections were treated with 3% hydrogen peroxide for 20 min, then blocked in 5% *(v/v)* goat serum for 1 h at room temperature. Sections were incubated with primary antibodies against claudin 2 (Abcam, Cambridge, United Kingdom; #ab53032; 1/100), claudin 3 (Abcam, Cambridge, UK; #ab15102, 1/100), PCNA (Santa Cruz Technology, Dallas, TX, USA; #sc7907, 1/200), cleaved caspase 3 (Cell Signalling Technology, Danvers, MA, USA; #9661; 1/150), CD4 (eBioscience, San Diego, CA, USA; #14-9766-82; 1/100), CD8ɑ (eBioscience, San Diego, CA, USA; 14-0808-82; 1/150), and FOXP3 (eBioscience; San Diego, CA, USA; 14-5773-80; 1/100) diluted in 5% (*v*/*v*) goat serum, overnight at 4 °C. Sections were then incubated with HRP secondary antibodies or biotinylated secondary antibodies (Avidin Biotin Complex ABC-kit; Vector Laboratories, Burlingame, CA, USA) as per the manufacturer’s instructions. Sections were developed using a Diaminobenzidine (DAB) substrate Chromogen System (Dako, Brüsseler Str., Berlin Germany) and counterstained with haematoxylin. Slides were imaged with an Aperio Slide Scanner (Leica Biosystems, Melbourne, Australia) and quantified using Aperio ImageScope v12.4.0.5043 software (Leica Microsystems Pty Ltd., Wetzlar, Germany). For histological analyses, the entire colons presented in “Swiss rolls” were analysed for each animal using algorithms by Aperio ImageScope, thereby preventing the bias associated with analysing different fields of view. An algorithm integrating pixels for positive stains across the entire cell was adopted for PCNA, claudin 2 and 3, and positive nuclei for cleaved caspase 3 and FOXP3 respectively, whereas CD4 and CD8 positivity was analysed using a cell membrane-based algorithm.

### 2.8. Multiplex ELISA

Cytokine assessment of mouse colonic tissues was performed in duplicate using ProcartaPlex Mouse Custom 15-plex (Cat #PPX-15-MXH6AJC; Lot #229576-000) and Procarta Mouse Basic Kit (Cat #EPX010-20440-901; Lot #231273-000) by Crux Biolabs (Melbourne, Australia). Upper and lower limits of detection for each cytokine are listed in the supplementary data (Table S2).

### 2.9. Statistical Analysis

Data are presented as mean ± SEM and were analysed using GraphPad Prism, version 8 (GraphPad, San Diego, CA, USA). The Kolmogorov–Smirnov test was used to assess the normality of distribution and the F test was used to compare variances. Differences between the two groups were analysed by unpaired Student’s *t*-test for equal variances and Welch’s *t*-test for unequal variances. For multiple comparisons, statistical analysis was performed using a one-way ANOVA with Tukey’s multiple comparisons test. Experiments with repeated measures data were analysed using a mixed effects model and adjusted for multiple comparisons with Bonferroni correction on GraphPad Prism. A *p*-value < 0.05 was considered to be statistically significant.

## 3. Results

### 3.1. Mice with Reduced STAT3 Activity Are Highly Susceptible to DSS-Induced Colitis In Vivo

In the present study, two mouse models were utilised to examine the significance of IL-6ST/gp130-dependent activation of STAT3 in intestinal barrier function during colitis. The IL-6ST/gp130 receptor is crucial for signal transduction of the IL-6 cytokine family, forming a receptor complex with the cytokine-bound ɑ receptor subunit to facilitate association of JAK/STATs and induce downstream cellular transcription. The GP130^∆STAT/+^ mouse model harbours a COOH-terminal truncation mutation in the IL-6ST/gp130 receptor that removes STAT1 and STAT3 binding sites, thus reducing IL-6ST/gp130-dependent STAT1 and STAT3 activation [22]. Heterozygous mice were used in this study, since homozygous GP130^∆STAT/∆STAT^ mice develop a range of abnormalities including gastrointestinal ulceration, degenerative joint disease, impaired bone growth [23], dysregulation of haematopoiesis [18], and failure of uterine implantation [22]. By contrast, heterozygous GP130^∆STAT/+^ mice confer an approximately 50% reduction in STAT3 activation, as determined by the reduced abundance of the active phosphorylated isoform of STAT3 (pSTAT3) (Supplementary Figure S1A). GP130^∆STAT/+^ mice are phenotypically normal with no signs of spontaneous gastrointestinal pathology under normal tissue homeostasis (Supplementary Figure S2A,B).

The second CAGs-rtTA;shStat3 mouse model, referred to as shStat3 hereafter, was generated to carry a GFP-linked, doxycycline (dox)-inducible *Stat3* short hairpin RNA for the reversible silencing of *Stat3* and has been characterised previously [19]. Two different controls were used to complement the dox-treated shStat3 cohort. The first experimental control included shStat3 mice that received a standard chow control diet (shStat3 chow), which does not induce the activity of the short hairpin RNA. In addition, we also employed dox-treated CAGs-rtTA;shLuc (referred to as shLuc) transgenic mice, which harbour a GFP-linked irrelevant hairpin to account for any non-specific effects from the short hairpin RNA. Dox treatment resulted in *Stat3* knockdown by 80% in multiple organs and cell types including the gastrointestinal tract, while *Stat1* expression remained unaltered [19]. Immunohistochemistry confirmed a reduction in the expression of pSTAT3 in shStat3 mice after doxycycline treatment, but not in shStat3 chow and dox-treated shLuc controls (Supplementary Figure S1D). Short hairpin expression or *Stat3* silencing did not have an effect on their overall phenotype [19] or intestinal morphology under normal tissue homeostasis (Supplementary Figure S2C,D).

**Figure 1:**
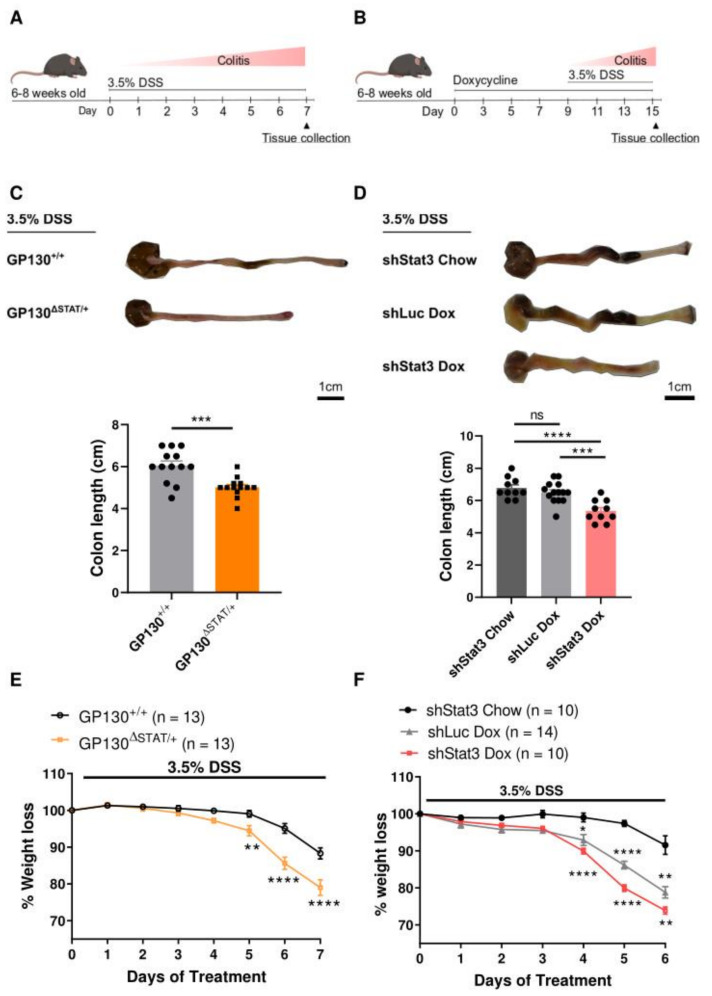
Mice with reduced STAT3 activity are highly susceptible to chemically induced colitis: (**A**) GP130^∆STAT/+^ and GP130^+/+^ controls were challenged with 3.5% dextran sulfate sodium (DSS) to induce colitis (*n* = 13 for each genotype). (**B**) shStat3 and shLuc controls either received standard chow or doxycycline diet for 9 days to induce short hairpin-driven *Stat3* silencing prior to DSS treatment (*n* = 10–14 for each experimental group). Experimental endpoint was determined by a 20% loss of initial weight. (**C**,**D**) Representative images and measurements of colon lengths from each cohort after DSS treatment. (**E**,**F**) Percentage of body weight loss during exposure to 3.5% DSS. Each symbol represents the data from an individual mouse and pooled across 3 independent experiments, mean ± SEM. Data were checked for normal distribution with Kolmogorov–Smirnov test and statistical analysis was performed using (**C**) unpaired Student’s *t*-test; (**D**) one-way ANOVA with Tukey’s multiple comparisons test; (**E**,**F**) mixed effects analysis with Bonferroni correction. * *p* < 0.05; ** *p* < 0.01; *** *p* < 0.001, **** *p* < 0.0001.

To investigate the functional role of IL-6ST/gp130-dependent STAT3 during colitis development, we induced acute colonic injury and inflammation by administration of DSS via drinking water. GP130^∆STAT/+^ and GP130^+/+^ control mice were challenged with 3.5% DSS (Figure 1A). shStat3 mice received dox pre-treatment for 9 days to induce *Stat3* knockdown, followed by treatment with 3.5% DSS (Figure 1B). We first compared epithelial STAT3 activation from dissected colons using Western blots. Minimal levels of pSTAT3 were observed in untreated control mice, whereas STAT3 phosphorylation was significantly induced in colonic tissue upon challenge with DSS (Supplementary Figure S1A–C). This suggests that STAT3 activation is stimulated upon damage to the intestinal epithelium. Strikingly, a reduction in STAT3 activity in GP130^∆STAT/+^ and dox-treated shStat3 mice resulted in significant colon shortening upon DSS treatment, which is a macroscopic marker for colonic injury (Figure 1C,D). As cytokines of the IL-6 and IL-10 families, including IL-6, IL-10, and IL-22, are implicated in supporting intestinal wound healing and immune regulation during colitis [12,24], we evaluated the production of these cytokines upon DSS treatment. In comparison to IL-10 and IL-22, we observed that IL-6 was the most abundantly produced cytokine during DSS-induced colitis development in wild-type mice as well as in mice with reduced STAT3 activity (Supplementary Figure S3A–G). The abundance of IL-6 was also significantly higher in GP130^∆STAT/+^ and dox-treated shStat3 mice after DSS treatment, suggesting a compensatory response in IL-6 production upon the reduction in STAT3 activity (Supplementary Figure S3B,E). Together, these findings demonstrate that IL-6ST/gp130/STAT3 activation plays a prominent role in regulating intestinal barrier function during colitis.

Compared to wild-type mice, which only developed mild colitis, a reduction in STAT3 activity resulted in significant weight loss in GP130^∆STAT/+^ and dox-treated shStat3 mice after DSS treatment (Figure 1E,F). Weight loss was also observed in dox-treated shLuc controls, but an additive effect was observed in dox-treated shStat3 mice. Interestingly, GP130^∆STAT/+^ mice with a 50% reduction in IL-6ST/gp130-dependent STAT3 activity succumbed to illness by day 7 in contrast to 6 days in dox-treated shStat3 mice (80% knockdown of *Stat3*). Likewise, mice with complete genetic *Stat3* ablation driven by the RosaCre^ERT2^ promoter (RosaCre^ERT2^;Stat3^fl/fl^) reached the experimental endpoint by day 4 (Supplementary Figure S4), indicating that the levels of cellular STAT3 activity inversely correlate with colitis severity.

### 3.2. Reduction in STAT3 Activity Decreases Intestinal Barrier Function and Compromises Barrier Integrity

**Figure 2:**
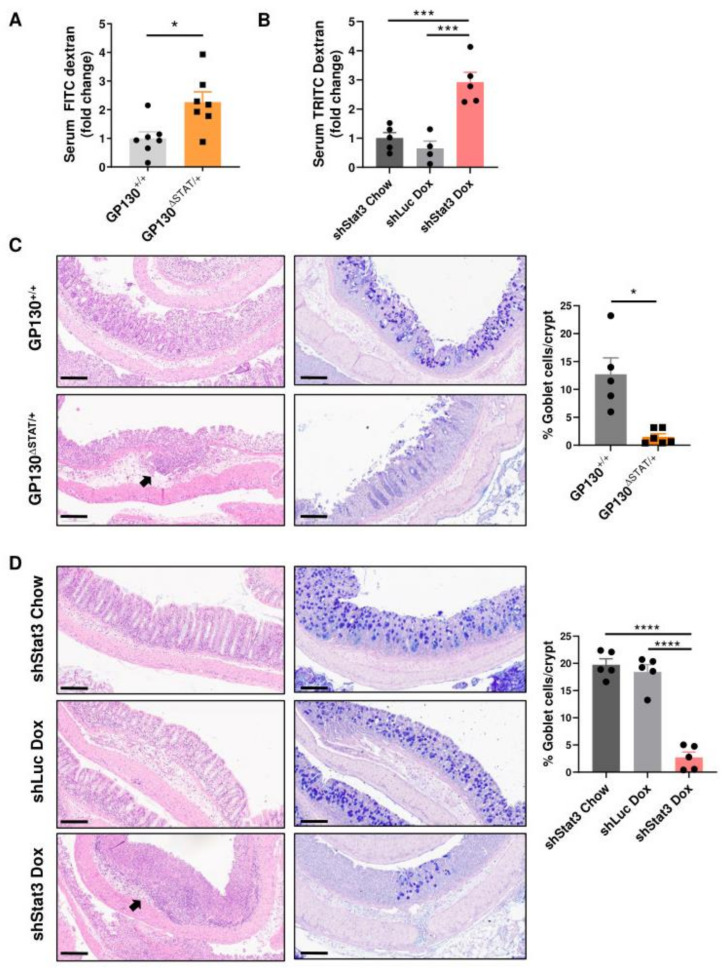
Reduction in STAT3 activity decreases intestinal barrier function and compromises barrier integrity: (**A**,**B**) FITC- or TRITC-labelled dextran was orally gavaged to mice at the end of the acute DSS challenge. Serum was collected 4 h later and fluorescence intensity was quantified as a measure of intestinal permeability (*n* = 4–7 per experimental group). (**C**,**D**) Representative images of H&E or PAS/Alcian blue-stained colon sections from GP130^+/+^, GP130^∆STAT/+^, shStat3 Chow, shLuc controls, and shStat3 mice treated with doxycycline (*n* = 5–6 per experimental group). Arrows indicate immune cell aggregates. Scale bar: 200 µm. Each symbol represents the data from an individual mouse and pooled across 3 independent experiments, mean ± SEM. Data were checked for normal distribution with Kolmogorov–Smirnov test and statistical analysis was performed using (**A**) unpaired Student’s *t*-test, **(B**,**D**) one-way ANOVA with Tukey’s multiple comparisons test, or (**C**) unpaired Student’s *t*-test with Welch correction. * *p* < 0.05; ** *p* < 0.01; *** *p* < 0.001; **** *p* < 0.0001.

In order to determine if STAT3 activity is required for sustaining intestinal barrier function in colitis, an in vivo intestinal permeability test was performed using oral administration of an indigestible, fluorescently labelled dextran and the fluorescence signal present in blood serum was measured. Compared to the GP130^+/+^ control, GP130^∆STAT/+^ demonstrated a 2.3-fold increase in intestinal permeability after DSS treatment (Figure 2A). To prevent spectral overlap between GFP expression and FITC-dextran, TRITC-dextran was adapted in the shStat3 and shLuc cohort. Similar to the GP130^∆STAT/+^ mice, dox-treated shStat3 mice displayed a 3-fold increase in intestinal permeability compared to their respective controls after DSS treatment (Figure 2B). Hence, the reduction in STAT3 activity in both mouse models significantly compromised intestinal barrier function in the context of colitis. Additionally, histopathological examination of the dissected colons was performed to assess the epithelial integrity in the two mouse models of reduced STAT3 activity after DSS treatment. GP130^+/+^, shStat3 chow and shLuc dox control mice displayed mild intestinal inflammation. In contrast, GP130^∆STAT/+^ and dox-treated shStat3 mice showed pronounced damage of the intestinal epithelium, characterised by severe crypt loss and increased immune cell infiltration into the colonic submucosa (Figure 2C,D, left panels). As mucus-producing goblet cells are essential for building a protective mucosal barrier to prevent the infiltration of bacteria and other inflammatory agents [25], PAS/Alcian blue staining was performed to quantify the number of goblet cells per crypt. We observed a substantial loss of goblet cells in the inflamed colonic crypts of both mouse models with reduced STAT3 activity (Figure 2C,D, right panels). These results demonstrate that STAT3 activity is critical for the maintenance of barrier integrity and associated intestinal permeability during the development of colitis.

### 3.3. Reduction in STAT3 Activity Decreases Epithelial Proliferation while Exacerbating Inflammatory Responses

**Figure 3:**
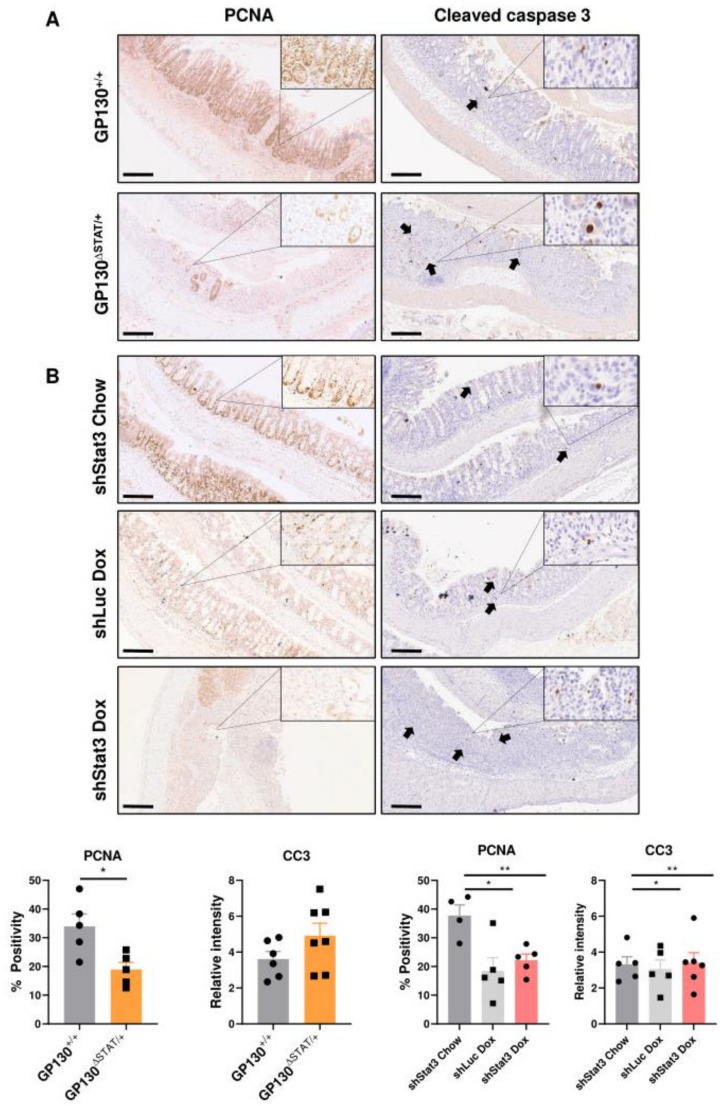
Impaired intestinal wound healing in mice with reduced STAT3 activity: (**A**) Immunohistochemistry assessment of proliferation (PCNA: proliferating cell nuclear antigen) and (**B**) apoptosis (CC3: cleaved caspase 3) of colonic sections from respective groups at the experimental endpoint (*n* = 4–7 per experimental group). Scale bar: 200 µm. Data were checked for normal distribution with Kolmogorov–Smirnov test and statistical analysis was performed using unpaired Student’s *t*-test or one-way ANOVA with Tukey’s multiple comparisons test. * *p* < 0.05; ** *p* < 0.01; *** *p* < 0.001; **** *p* < 0.0001.

Intestinal wound healing in response to DSS-induced injury is dependent on survival and the regenerative capacity of IECs. The expression of the proliferation marker PCNA was, therefore, evaluated from the dissected colons at the experimental endpoint. We demonstrated that GP130^∆STAT/+^ and shStat3 mice both exhibited a significant reduction in PCNA expression, while the number of cleaved caspase-3 positive apoptotic cells remained unchanged after DSS treatment (Figure 3A,B). These data indicate that STAT3 activity is required to sustain epithelial proliferation during colitis.

**Figure 4:**
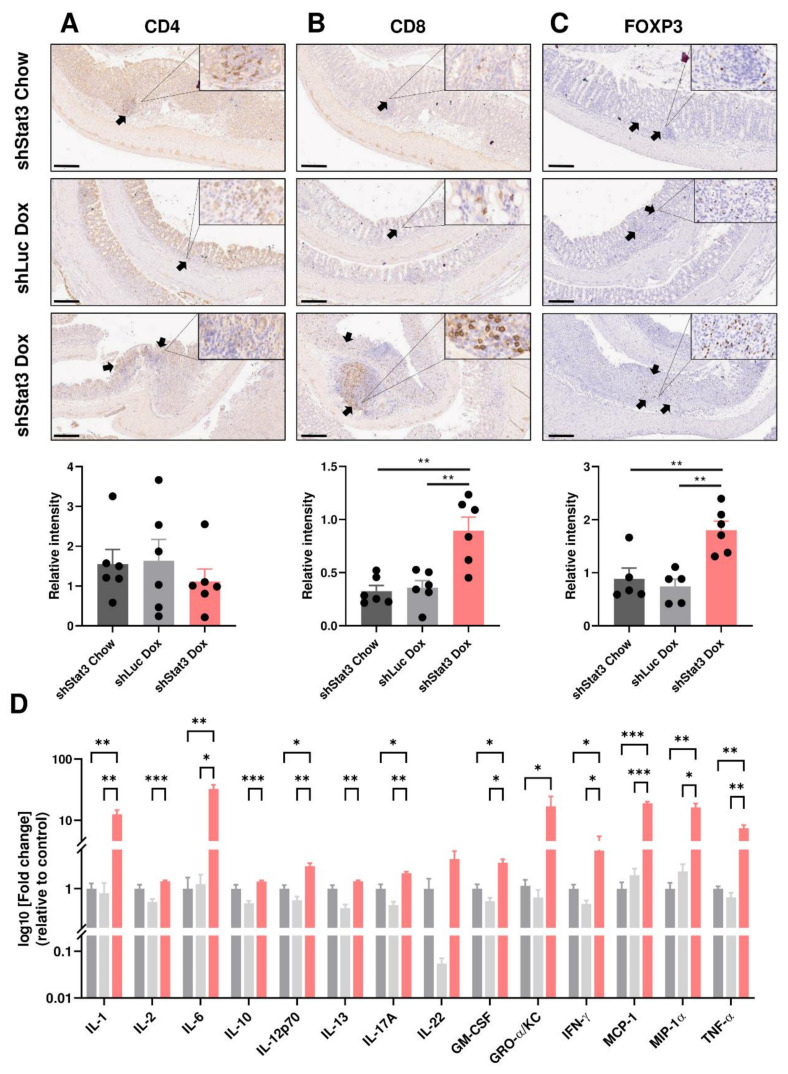
Stat3 knockdown increases immune cell infiltration and elevates production of cytokines and chemokines in colonic tissue. Immunohistochemistry assessment of: (**A**) CD4; (**B**) CD8; and (**C**) FOXP3 of colons harvested from shStat3 Chow, shStat3 Dox, and shLuc at the experimental endpoint (*n* = 5–6 per experimental group). Scale bar: 200 µm; (**D**) Cytokine and chemokine levels from colonic tissues harvested from shStat3 Chow, shStat3 Dox, and shLuc at the experimental endpoint as analysed by multiplex cytokine array. The relative fold change of each cytokine in dox-treated shStat3 and shLuc group was compared to that of the shStat3 Chow control. Data represent mean ± SEM. Data were checked for normal distribution with Kolmogorov–Smirnov test and statistical analysis was performed using one-way ANOVA with Tukey’s multiple comparisons test. * *p* < 0.05; ** *p* < 0.01; *** *p* < 0.001; **** *p* < 0.0001.

Given the more severe phenotype and impaired barrier function observed in mice with *Stat3* knockdown, we focused on identifying the different cell types in the immune aggregates observed in the shStat3 cohort following DSS treatment. Immunohistochemical analysis revealed that knockdown of *Stat3* resulted in increased infiltration of CD8^+^ and FOXP3^+^ cells, but not CD4^+^ cells, into the colonic epithelium in response to DSS treatment (Figure 4A–C). Compared to shStat3 chow and shLuc controls, the colonic expression of the pro-inflammatory cytokines, including IL-1, IL-6, IL-17A, IFN-γ, and TNF-α, was significantly upregulated in dox-treated shStat3 mice. This coincided with increased levels of the inflammatory KC/CXCL1, MCP-1/CCL2, and MIP-1ɑ/CCL3 chemokines in colonic tissue (Figure 4D). These findings demonstrate that a reduction in STAT3 activity increases the accumulation of infiltrating immune cells and induces the production of cytokines that are frequently upregulated in mouse models of IBD [26].

### 3.4. Reduction in STAT3 Activity Compromises Expression of Antimicrobial Genes and Tight Junction Proteins

**Figure 5:**
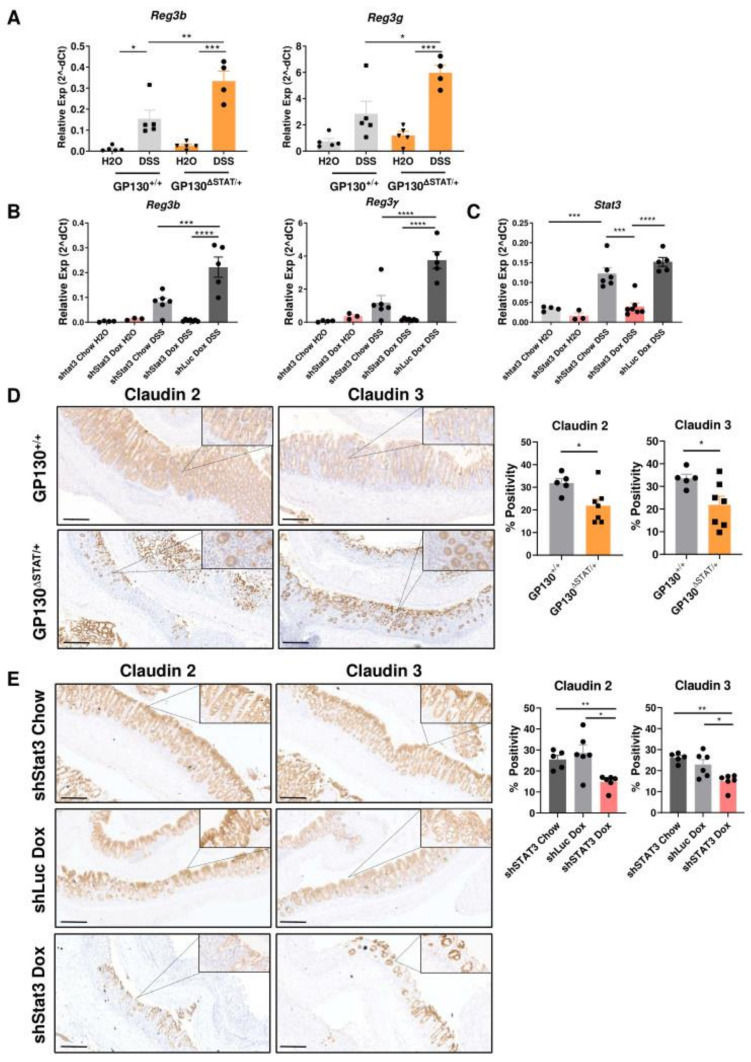
Reduction in STAT3 activity impairs expression of antimicrobial genes and tight junction proteins. The gene expression of *Reg3b* and *Reg3g* (**A**,**B**) and *Stat3* (**C**) was analysed in colonic lysates from each cohort using real-time quantitative PCR in technical duplicate (*n* = 3–6 per experimental group). Data represent relative expression normalised against the housekeeping gene *Gapdh,* with mean ± SEM. Immunohistochemistry analysis of claudin 2 and claudin 3 of colons harvested from (**D**) GP130^+/+^ and GP130^ΔSTAT/+^ and (**E**) shStat3 Chow, shStat3 Dox, and shLuc at experimental endpoint (*n* = 5–7 per experimental group). Data represent mean ± SEM. Data were checked for normal distribution with Kolmogorov–Smirnov test and statistical analysis was performed using (**A**,**B**,**E**) one-way ANOVA with Tukey’s multiple comparisons test or (**D**) unpaired Student’s *t*-test. Scale bar: 200 µm. * *p* < 0.05; ** *p* < 0.01. *Reg3*: Regenerating islet-derived protein-3.

The *Reg3* family antimicrobial genes have previously been identified as *Stat3* target genes through direct binding at the promoter region [17,27]. We therefore hypothesised that GP130^∆STAT/+^ and dox-treated shStat3 mice may exhibit a defective antimicrobial defence. The expression of *Reg3b* and *Reg3g* was measured using real-time quantitative PCR. Compared to untreated controls, the expression of these antimicrobial genes was markedly upregulated after DSS treatment in both mouse models. The expression of *Reg3b* and *Reg3g* significantly increased by two-fold in DSS-treated GP130^∆STAT/+^ mice compared to GP130^+/+^ controls (Figure 5A). As the GP130^∆STAT/+^ model only partially reduces phosphorylation and activation of STAT3, the observed induction of *Reg3b* and *Reg3g* mRNA transcript was likely mediated by residual STAT3 signalling. Furthermore, induction of the short hairpin RNA with dox treatment resulted in an 80% knockdown in *Stat3* mRNA transcript in the colonic tissue of shStat3 mice, but not in chow-treated or shLuc controls (Figure 5C). This *Stat3* knockdown led to a >80% decrease in the transcript levels of *Reg3b* and *Reg3g* in dox-treated shStat3 mice upon DSS challenge (Figure 5B). These data indicate that STAT3 may confer a protective effect against pathogens by controlling bacterial growth and infection via transcriptional regulation of antimicrobial genes during colitis development.

The claudin family of proteins constitutes the tight junctions between adjacent IECs to regulate passage of ions, molecules, and barrier permeability. Shifts in the expression of tight junction proteins can result in barrier dysfunction and invasion of pathogenic bacteria across the intestinal epithelium, which are associated with the pathogenesis of IBD [28,29,30]. While previous studies have demonstrated that the STAT3 activators IL-6 and IL-22 can regulate the expression of claudin 2 to regulate intestinal permeability in vitro [31,32,33], whether this effect is observed in vivo is yet to be investigated. Western blot analysis showed that the expression of claudin 2 and claudin 3 was comparable between GP130^+/+^ and GP130^∆STAT/+^ or shStat3 and control mice under normal homeostasis (Supplementary Figure S1C. Strikingly, the expression of these proteins was profoundly impaired in mice with reduced STAT3 expression upon DSS challenge (Supplementary Figure S1A,B). Using immunohistochemistry, we further confirmed that the loss of crypt architecture observed in GP130^∆STAT/+^ and dox-treated shStat3 mice correlated with defective expression of claudin 2 and 3 (Figure 5D,E). Collectively, our findings suggest that a reduction in IL-6ST/gp130-dependent STAT3 activity results in dysregulated expression of tight junction proteins, thereby impairing barrier function to increase susceptibility to DSS-induced colitis.

## 4. Discussion

To elucidate the importance of signal transduction emanating from the shared IL-6 family cytokine receptor IL-6ST/gp130 through STAT3 in the pathogenesis of IBD, our study aimed to define the mechanisms by which STAT3 regulates intestinal barrier integrity during DSS-induced colitis. A reduction in STAT3 activity did not affect intestinal morphology under steady state conditions. In comparison to the other STAT3-activating cytokines profiled, IL-6 showed the most prominent increase after DSS treatment. This finding is consistent with our previous observation that IL-6-deficient mice showed similar susceptibility to acute DSS injury as homozygous GP130^∆STAT^ mice [18], further reinforcing the significance of IL-6 in mediating IL-6ST/gp130-dependent STAT3 signalling in colitis development. In the present study, we used two complementary mouse models to formally align the cellular mechanisms involved in colitis development to STAT3 activity. A reduction in STAT3 activity resulted in more severe colitis and impaired epithelial proliferation. The GP130^∆STAT/+^ and shStat3 mouse models recapitulated previous studies where IEC-specific *Stat3* ablation resulted in enhanced susceptibility to colitis with increased weight loss, histological injury, and inflammation in response to DSS [11,16]. Our data indicate that IL-6-mediated IL-6ST/gp130 signalling drives STAT3 activation, by which STAT3 modulates intestinal permeability through the expression of tight junction proteins during the development of colitis. Additionally, our results demonstrate that a reduction in STAT3 activity in the GP130^∆STAT/+^ and shStat3 mouse models impaired antimicrobial gene expression.

In the context of IBD, IL-6 is reported to be produced by a variety of cell types, including lamina propria mononuclear cells and intraepithelial lymphocytes [34,35]. IL-6 and STAT3 together form a tightly regulated positive feedback loop, whereby STAT3 regulates IL-6 expression through binding to the *IL-6* promoter [36]. Previous studies in a mouse model of colitis have demonstrated that mice with IEC-specific deletion of *Stat3* display increased IL-6 expression [11]. In our study, we have observed increased production of IL-6 in both GP130^∆STAT/+^ and dox-treated shStat3 mice after DSS treatment, which is likely to be attributed by a compensatory response mediated via residual STAT3 activity.

Previous studies have demonstrated that STAT3 confers a protective effect during acute colitis by sustaining proliferation and promoting a wound healing response [11,16]. We further demonstrate that STAT3 is critical for promoting intestinal integrity, as reduced STAT3 activity is associated with barrier breakdown and damage in the protective mucosal barrier as reflected by goblet cell loss. Goblet cell loss may further exacerbate inflammation and predispose these mice to bacterial translocation to the lumen. Furthermore, damage to the colonic epithelium dramatically exacerbated inflammation and increased infiltration of CD8^+^ T cells and FOXP3^+^ regulatory T cells to the site of injury. Interestingly, we did not observe an increase in CD4^+^ helper T cells in our study. The increased proportion of CD8^+^ T cells and FOXP3^+^ might implicate a regulatory role of CD8^+^ FOXP3^+^ Tregs during the development of IBD in mice with reduced STAT3 activity. In accordance with this, Sun et al. demonstrated that a proportion of CD8^+^ FOXP3^+^ Tregs is upregulated in mice during the late phase of DSS-induced colitis [37]. In addition, we have observed increased production of multiple pro-inflammatory cytokines and chemokines in mice with *Stat3* knockdown. A previous study has demonstrated that the production of cytokines such as IL-1ɑ, IL-6, IL-10, and IFN-γ are significantly reduced in mice with enhanced STAT3 activity during DSS-induced colitis [38]. Given that STAT3 is also responsible for inducing the secretion of immunosuppressive cytokines [36], the increased production of cytokines observed in our study may be the consequence of reduced STAT3 activity as well as an enhanced immune response to the site of injury.

Considering that STAT3 has been shown to drive the expression of genes regulating cell cycle, survival, and proliferation in IECs [39], we observed that a global reduction in STAT3 activity resulted in decreased epithelium proliferation, regeneration, and induced goblet cell loss in our mouse models. Although complete *Stat3* ablation in IECs increased the abundance of apoptotic IECs upon DSS treatment [16,17], partial reduction in STAT3 activity in GP130^∆STAT/+^ and shStat3 mice did not reveal significant differences in cleaved caspase 3-positive IECs. This may be attributed to residual STAT3 activity in these mouse models or due to the majority of apoptotic events taking place after 2–3 days of DSS treatment [40]; differences in caspase 3 may, therefore, not be captured at our experimental endpoints (6–7 days after treatment).

A biochemical mechanism by which IEC governs barrier function is through the production of antimicrobial peptides. The C-type lectin regenerating islet-derived protein III (Reg3) is well-known to exert bactericidal activity against Gram-positive as well as commensal bacteria [41]. While the Reg family of proteins is highly expressed in human IBD patients [42], a recent study has uncovered its contribution in promoting the colonisation of enteropathogens and prolonging infections [43]. Thus, the profound induction of *Reg3b* and *Reg3g* in GP130^ΔSTAT/+^ mice strongly implicates that IL-6ST/gp130/STAT3 signalling contributes to the regulation of host microbiome composition during colitis. Since Reg3 family antimicrobial peptides have been reported to shape the composition of the gut microbiota in metabolic diseases [44,45], our findings warrant further exploration into whether the modulation of STAT3 activity alters microbial communities in mouse models of IBD.

Tight junction proteins connect adjacent IECs to modulate intestinal permeability and barrier integrity. Expression of the tight junction protein claudin 2 is abundantly upregulated in human patients and mouse models of IBD [46]. However, current knowledge of the function of claudin 2 remains limited to its pore-forming effects in the intestinal epithelium. Studies by Wang et al. and Suzuki et al. demonstrated that IL-6 and IL-22 are able to regulate permeability via the JAK/STAT3 signalling pathway in a human colonic epithelial cell line [31,33]. Strikingly, overexpression of claudin 2 in transgenic mice and in a colonic epithelial cell line showed that claudin 2 offers protection against DSS-induced colitis and also altered the proportion of FOXP3^+^ regulatory T cells in injured colonic tissue [47]. In line with this observation, knockdown of *Stat3* in shStat3 mice reduced the expression of claudin 2 and increased the abundance of FOXP3^+^ cells. These findings reveal a complex relationship between STAT3 and claudin 2 in the regulation of intestinal homeostasis, immune activation, and inflammation. We therefore postulated that IL-6ST/gp130-dependent STAT3 activation may coordinate the expression of claudin 2 to facilitate immune cell trafficking to IECs during the pathogenesis of IBD. Similarly, downregulation of claudin 3 has been reported in patients with IBD [48]. Ahmad et al. have previously identified that claudin 3 is regulated by IL-6/gp130/STAT3 signalling in the colonic epithelium [49]. Expression of claudin 3 is also significantly lower in the colon of STAT3-deficient mice [50]. These studies collectively support the notion that STAT3 promotes intestinal integrity through the expression of claudin 2 and claudin 3. The reduction in STAT3 activity in both mouse models results in diminished expression of these tight junction proteins and ultimately resulted in barrier breakdown. Further studies are, therefore, necessary to address whether claudin 2 and 3 are direct targets of STAT3.

The therapeutic potential of harnessing the STAT3 signalling cascade as a treatment of IBD has been highlighted in previous reports. Hyperactivation of STAT3 as well as STAT1 in the GP130Y757F/Y757F mouse model confers resistance against DSS-induced epithelial injury [11,24,39]. Analysis of the compound GP130Y757F/Y757F mouse model with additional heterozygous deletion of either one *Stat3* or *Stat1* allele revealed that the protective effect is attributed to STAT3 but not STAT1 [11]. In line with this finding, Kobayashi et al. validated that STAT1 does not play a role in intestinal barrier function during the development of colitis [11,14]. The reduction in the capacity of the IL-6ST/gp130 receptor to activate STAT1 and STAT3 in our heterozygous GP130^∆STAT/+^ mice demonstrates that IL-6ST/gp130 receptor signalling is required for the maintenance of intestinal barrier function during colitis. Given that knockdown of Stat3 does not have an effect on Stat1 expression in shStat3 mice [19] and the similar disease phenotype in GP130^∆STAT/+^ and shStat3 mice after DSS treatment, these further support that STAT3, but not STAT1, confers protection against colitis. Furthermore, significant weight loss was observed in the dox-treated shLuc control group but not in the shStat3 chow control group, which may be partly due to the use of the antibiotic doxycycline to induce short hairpin activity in this mouse model. It is important to note, however, an exacerbated phenotype was only observed in dox-treated shStat3 mice in all other surrogate markers for DSS-induced acute colitis (colon length, intestinal permeability, cell morphology, goblet cell number, immune cell infiltration, production of cytokines), strongly arguing that the effects are mediated by reduced STAT3 activity rather than systemic effects of doxycycline treatment. Nonetheless, the observed weight loss in shLuc mice may be attributed to the use of doxycycline. Huang et al. have demonstrated that the use of antibiotics can induce microbial dysbiosis, which ultimately affects susceptibility to DSS-induced colitis in mice [51]. Likewise, the functional nature of doxycycline as a broad-spectrum antibiotic that targets a range of Gram-positive and Gram-negative bacteria may alter the microbial community, which results in weight loss in mice during IBD development. Importantly, dox-treated shLuc mice did not display colon shortening, which is a recognized hallmark of colitis. Doxycycline has also been shown to inhibit cell proliferation in a human colon cancer cell line [52], which is consistent with the reduced PCNA expression observed in dox-treated shLuc mice after DSS treatment. We therefore believe that a comparison between doxycycline-treated and doxycycline-naive shStat3 mice is a relevant comparison to assess the effects of reduced STAT3 activity on DSS-induced colitis.

**Figure 6:**
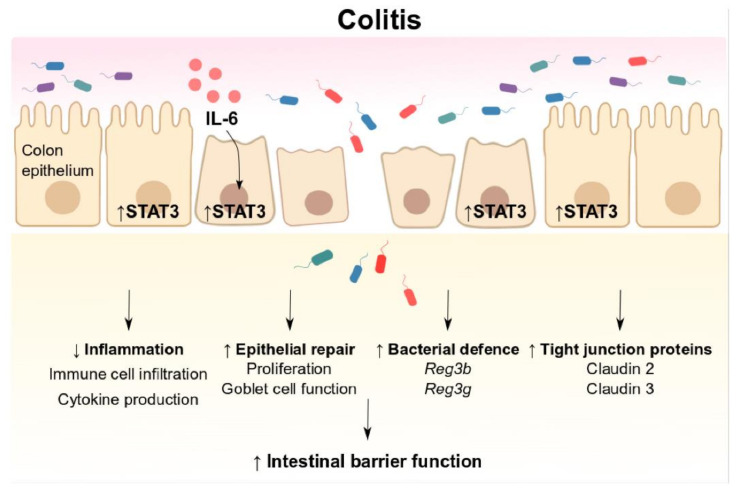
Schematic overview of IL-6ST/gp130/STAT3-mediated effects on intestinal barrier function during colitis. IL-6ST/gp130/STAT3 signalling is induced in the colonic epithelium during the development of colitis. STAT3 is central for regulating intestinal barrier function through several mechanisms: (i) STAT3 suppresses inflammation by regulating immune cell infiltration and cytokine production; (ii) promoting epithelial repair by enhancing proliferation and maintaining goblet cell function; (iii) enhancing bacterial defence through induction of the antimicrobial genes *Reg3b* and *Reg3g*; and (iv) regulating colonic permeability through the tight junction proteins, claudin 2 and claudin 3. IL: interleukin; STAT3: signal transducer and activator of transcription factor-3. *Reg3*: regenerating islet-derived protein-3.

Taken together, our study shows that the IL-6ST/gp130/STAT3 signalling pathway is central for protection against acute colitis (Figure 6). The high levels of IL-6 production associated with acute colitis are likely to be responsible for subsequent IL-6ST/gp130 signalling and downstream STAT3 activity. STAT3 maintains a barrier function through regulating epithelial regeneration, inflammation, and modulating defence against pathogens and intestinal permeability via tight junction proteins. While pharmacological inhibition of STAT3 has been an attractive target for anti-cancer therapies, it is possible that a systemic reduction in STAT3 activity may compromise intestinal barrier function and promote intestinal inflammation. Our study provides a rationale to exploit upstream activators of IL-6ST/gp130 to induce transient STAT3 activity to maintain barrier integrity and ameliorate colitis in IBD patients.

## Data Availability

The data that support the findings of this study are available from the corresponding authors upon reasonable request.

## Supplementary Materials

The following are available online at https://www.mdpi.com/2227-9059/9/2/187/s1. Supplementary Table S1: qPCR primers used in this study. Supplementary Table S2: Upper and lower limit of detection for multiplex cytokine profiling. Supplementary Figure S1: Comparison of protein expression under steady state and DSS challenge. Supplementary Figure S2: Reduction of STAT3 activity does not alter colon morphology under normal homeostasis. Supplementary Figure S3: Increased production of IL-6 cytokine during colitis development. Supplementary Figure S4: Complete *Stat3* ablation further increases susceptibility to chemically-induced colitis.
