# Supplementary material for: STAT3 Signalling via the IL-6ST/gp130 Cytokine Receptor Promotes Epithelial Integrity and Intestinal Barrier Function during DSS-Induced Colitis
Source: Biomedicines. 2021 Feb 12;9(2):187. doi: 10.3390/biomedicines9020187 (PMC7918037; doi:10.3390/biomedicines9020187)
Supplement: Supplementary file 1 [file biomedicines-09-00187-s001.pdf]

Supplementary Materials

Table S1. qPCR primers used in this study

Gene	Forward primer	Reverse primer
Reg3b	5′-CTCCTGCCTGATGCTCTTAT-3′	5′-TTGTTACTCCACTCCCATCC-3′
Reg3g	5′-TGCTGCTCTCCTGCCTGATG-3′	5′-ATAGGAGCCATAGGCACGGG-3′
Stat3	5′-AGAACCTCCAGGACGACTTTG-3′	5′-TCACAATGCTTCTCCGCATCT-3′
Gapdh	5′-TTGATGGCAACAATCTCCAC-3′	5′-CGTCCCGTAGACAAAATGGT-3′

Table S2. Upper and lower limit of detection for multiplex cytokine profiling

Analyte	MAX (pg/ml)	LOD (pg/ml)
**IL-1β**	4,350	1.06
**IL-2**	5,250	1.28
**IL-6**	19,500	4.76
**IL-10**	8,400	2.05
**IL-12p70**	6,550	1.6
**IL-13**	8,650	2.11
**IL-17A**	5,750	1.4
**IL-22**	40,400	9.86
**GM-CSF**	9,950	2.43
**GRO-α/KC**	5,950	1.45
**IFN-γ**	4,800	1.17
**MCP-1**	28,300	6.91
**MIP-1α**	1,400	0.34
**TNF-α**	11,700	2.86

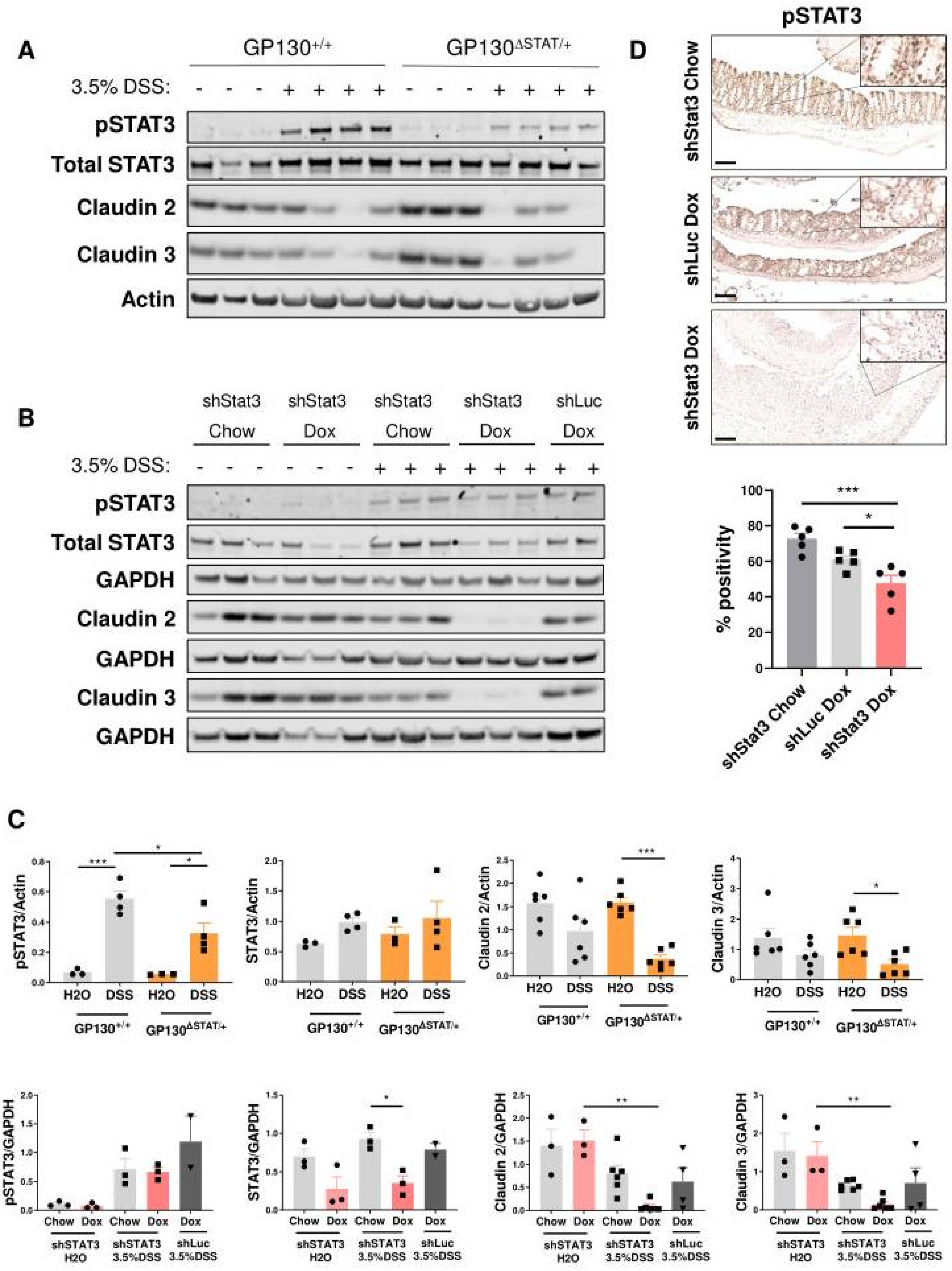

Supp figure S1. Comparison of protein expression under steady state and DSS challenge

Representative Western blots for indicated proteins on colonic lysates harvested from **(A)** GP130$^{+/+}$, GP130$^{\Delta STAT/+}$ or **(B)** shStat3 chow, shLuc controls and doxycycline-treated shStat3 before (N=3 per experimental group) or after treatment with 3.5% DSS (N=4-6 per experimental group). **(C)** Densitometric analysis of each respective protein with normalization to β-actin or GAPDH. **(D)** Immunohistochemical assessment of pSTAT3 of colonic sections from shStat3 Chow, dox-treated shStat3 and shLuc mice at experimental endpoint (N=5 per experimental group). Scale bar: 200μm. **(C-D)** Data analysis was performed using one-way ANOVA with Tukey's multiple comparisons test. *p<0.05; ** p<0.01; *** p<0.001.

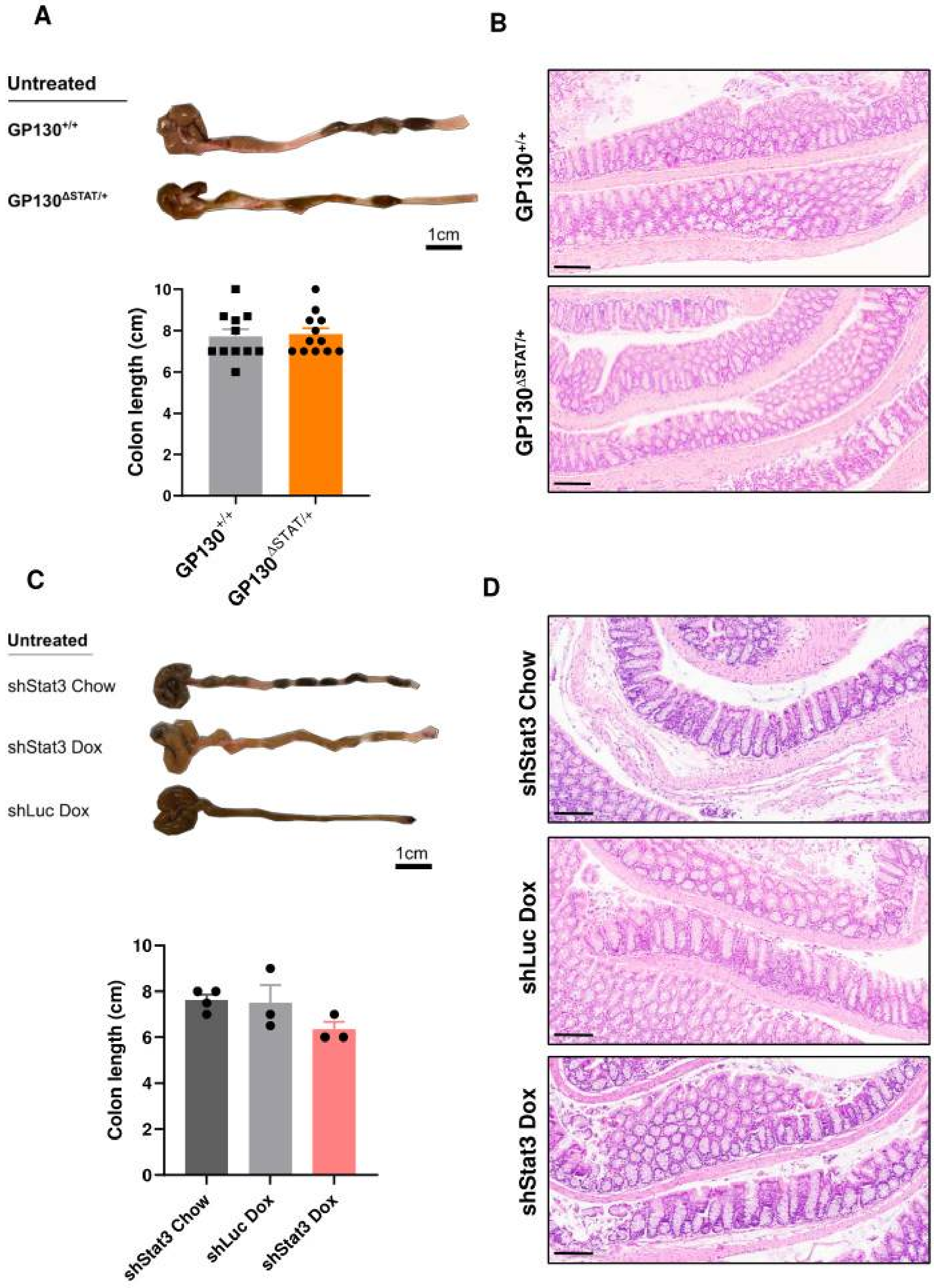

Supp figure S2. Reduction of STAT3 activity does not alter colon morphology under normal homeostasis

(A, C) Representative images and measurements of colon lengths of 6-8 week old mice from each cohort without DSS treatment. **(B, D)** H&E stained colon sections from GP130$^{+/+}$, GP130$^{\Delta STAT/+}$, shStat3 chow, shLuc controls and doxycycline-treated shStat3. Scale bar: 200μm.

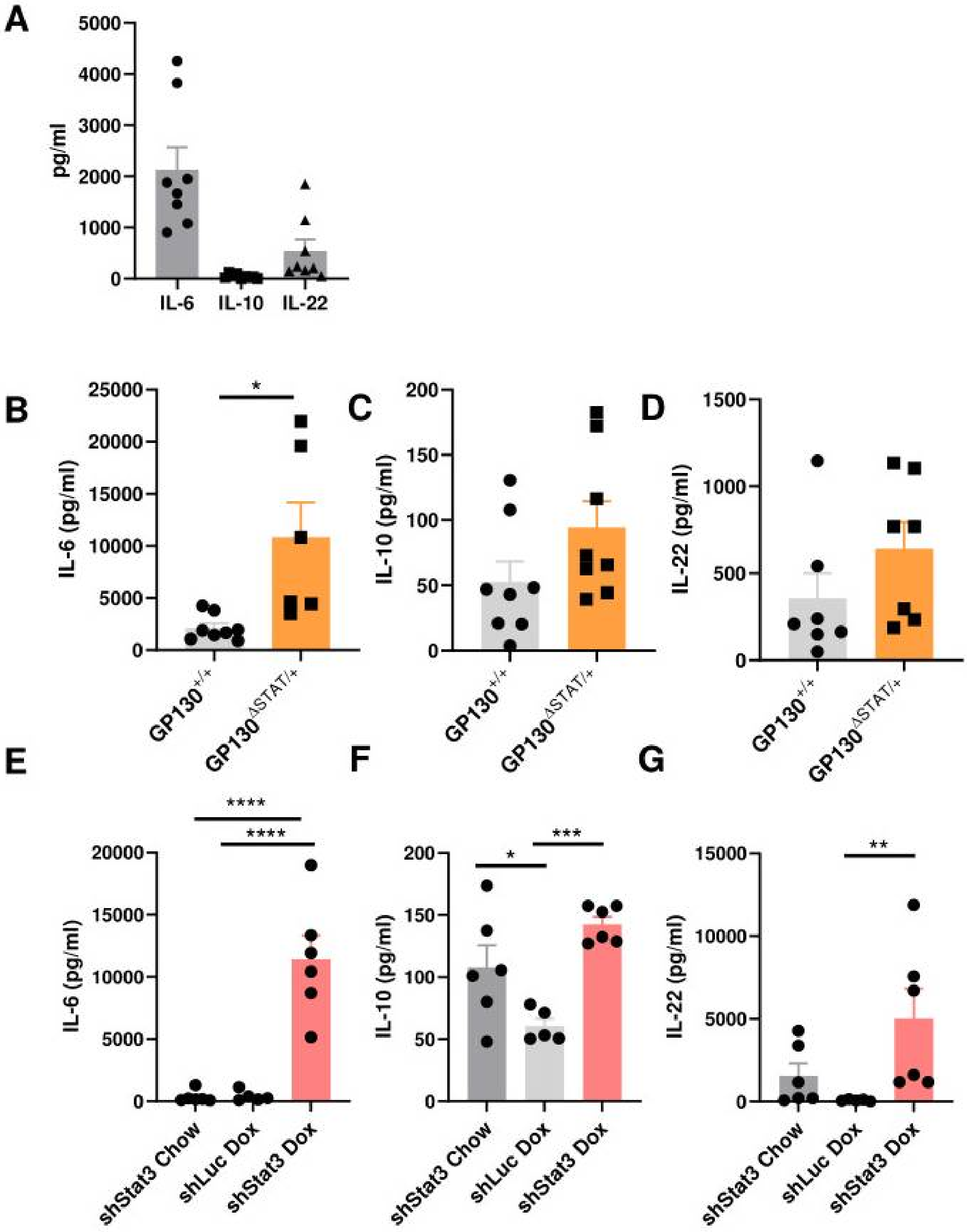

Supp figure S3. Increased production of IL-6 cytokine during colitis development

(A) Production of IL-6, IL-10 and IL-22 from colonic tissues harvested wild type mice upon exposure to 3.5% DSS. (B) Measurement of IL-6, IL-10 and IL-22 production by multiplex cytokine array in colonic tissues harvested from **(B)** $GP130^{+/+}$, $GP130^{\Delta STAT/+}$ or **(C)** shStat3 chow, shLuc controls and doxycycline-treated shStat3 after treatment with 3.5% DSS. Data represent mean ± SEM. Statistical analysis was performed using **(B)** unpaired Student's t-test with Welch correction, **(C-D)** unpaired Student's t-test or **(E-G)** one-way ANOVA with Tukey's multiple comparisons test. *$p<0.05$; ** $p<0.01$; *** $p<0.001$; **** $p<0.0001$.

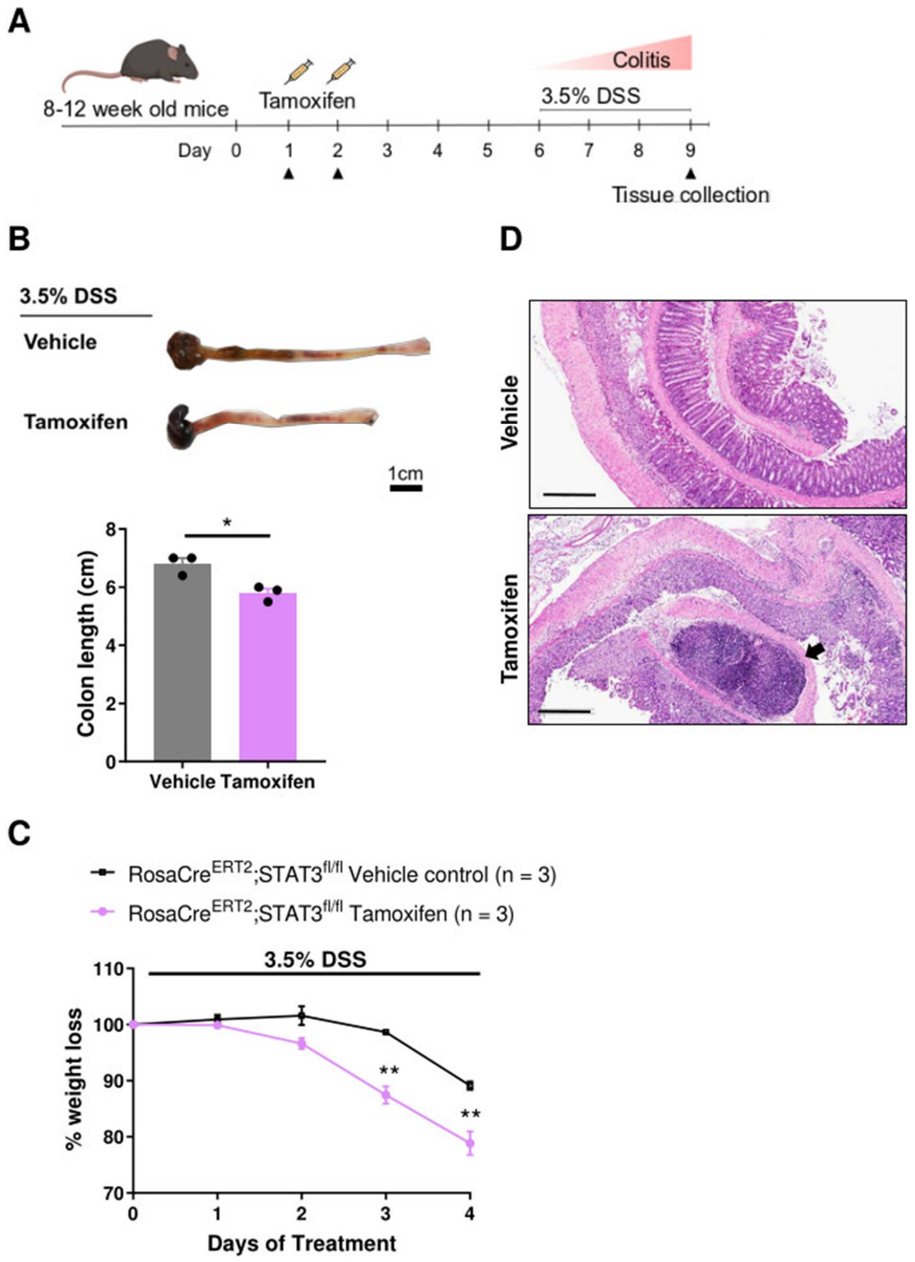

Supp figure S4. Complete *Stat3* ablation further increases susceptibility to chemically-induced colitis

(A) RosaCre^{ERT2};Stat3$^{fl/fl}$ mice received two doses of tamoxifen (5mg/25g) via oral gavage to induce activity of Cre recombinase and global *Stat3* deletion. Following 3 days of rest, mice were challenged with 3.5% DSS until experimental endpoint was reached. **(B)** Representative images and measurements of colon lengths harvested after 4 days of DSS treatment. **(C)** Percentage body weight loss during exposure to 3.5% DSS. **(D)** Representative images of H&E stained colon sections from vehicle or tamoxifen-treated RosaCre^{ERT2};Stat3$^{fl/fl}$ mice after exposure to DSS. Arrow indicates immune cell aggregates. Scale bar: 200µm. Statistical analysis was performed using **(B)** unpaired Student's t-test or **(C)** **mixed effects analysis with Bonferroni correction**. *p<0.05; ** p<0.01.
